# Metabolic syndrome related gene signature predicts the prognosis of patients with pancreatic ductal carcinoma. A novel link between metabolic dysregulation and pancreatic ductal carcinoma

**DOI:** 10.1186/s12935-021-02378-w

**Published:** 2021-12-20

**Authors:** Weiyang Cai, Wenming Bao, Shengwei Chen, Yan Yang, Yanyan Li

**Affiliations:** 1 Department of Ultrasound, Second Affiliated Hospital and Yuying Children’s Hospital of Wenzhou Medical University, 109 Xueyuanxi Road, Wenzhou, 325000 Zhejiang People’s Republic of China; 2 Department of Gastroenterology and Hepatology, The First Affiliated Hospital of Wenzhou Medical University, Wenzhou, China; 3 Department of Hepatobiliary Surgery, The First Affiliated Hospital of Wenzhou Medical University, Wenzhou, Zhejiang China; 4 Department of Nephrology, The People’s Hospital of Yuhuan, The Yuhuan Branch of The First Affiliated Hospital of Wenzhou Medical University, Yuhuan, China

**Keywords:** Metabolism, Pancreatic ductal adenocarcinoma, Immunity, CIBERSORT, GMPS

## Abstract

**Background:**

Pancreatic cancer is one of the most common malignancies worldwide. In recent years, specific metabolic activities, which involves the development of tumor, caused wide public concern. In this study, we wish to explore the correlation between metabolism and progression of tumor.

**Methods:**

A retrospective analysis including 95 patients with pancreatic ductal adenocarcinoma (PDAC) and PDAC patients from The Cancer Genome Atlas (TCGA), the International Cancer Genome Consortium (ICGC), and The Gene Expression Omnibus (GEO) database were involved in our study. Multivariate Cox regression analysis was used to construct the prognosis model. The potential connection between metabolism and immunity of PDAC was investigated through a weighted gene co-expression network analysis (WGCNA). 22 types of Tumor-infiltrating immune cells (TIICs) between high-risk and low-risk groups were estimated through CIBERSORT. Moreover, the potential immune-related signaling pathways between high-risk and low-risk groups were explored through the gene set enrichment analysis (GSEA). The role of key gene GMPS in developing pancreatic tumor was further investigated through CCK-8, colony-information, and Transwell.

**Results:**

The prognostic value of the MetS factors was analyzed using the Cox regression model, and a clinical MetS-based nomogram was established. Then, we established a metabolism-related signature to predict the prognosis of PDAC patients based on the TCGA databases and was validated in the ICGC database and the GEO database to find the distinct molecular mechanism of MetS genes in PDAC. The result of WGCNA showed that the blue module was associated with risk score, and genes in the blue module were found to be enriched in the immune-related signaling pathway. Furthermore, the result of CIBERSORT demonstrated that proportions of T cells CD8, T cells Regulatory, Tregs NK cells Activated, Dendritic cells Activated, and Mast cells Resting were different between high-risk and low-risk groups. These differences are potential causes of different prognoses of PDAC patients. GSEA and the protein–protein interaction network (PPI) further revealed that our metabolism-related signature was significantly enriched in immune‐related biological processes. Moreover, knockdown of GMPS in PDAC cells suppressed proliferation, migration, and invasion of tumor cells, whereas overexpression of GMPS performed oppositely.

**Conclusion:**

The results shine light on fundamental mechanisms of metabolic genes on PDAC and establish a reliable and referable signature to evaluate the prognosis of PDAC. GMPS was identified as a potential candidate oncogene with in PDAC, which can be a novel biomarker and therapeutic target for PDAC treatment.

**Supplementary Information:**

The online version contains supplementary material available at 10.1186/s12935-021-02378-w.

## Introduction

Pancreatic cancer is considered as one of the most devastating malignancies worldwide [1]. It is the fourth leading cause of cancer-related mortality in the US and is expected to become the second most common cause of cancer-related death in the US by 2030 [2]. Epidemiological characteristics of pancreatic cancer contain insidious onset, invasive fast-growing, high recurrence rate and fatality [3]. Among them, pancreatic ductal adenocarcinoma (PDAC) accounts for > 85% of all pancreatic cancer cases [4]. Despite the great progress in diagnoses, therapy methods and surgical managements of PDAC recently, the long-term survival rate of patients with PDAC is still very low. The 5-year overall survival rate of patients with PDAC is still less than 10%, which remains static since 1960s [5]. Previous studies have demonstrated that risk factors for PDAC include age, obesity, Diabetes Mellitus (DM), smoking, long-term alcohol consumption, family history, etc. [6, 7]. Increased body index (BMI) and long-term DM is reported to be associated with the development of PDAC [8–10].

In recent years, the importance of reprogrammed metabolism in cancer has received great attention [11]. The changes in cell metabolism contribute to the tumor initiation and progression. Specific metabolic activities can be involved in the transformation process or support the biological process which participates in tumor growth [12]. Plenty of studies have revealed that metabolism played an important role in the development of PDAC. The alterations of metabolism can promote the initiation and metastasis of PDAC by genetic control [13–15]. PDAC cells possess extensively reprogrammed metabolism including glutamine-dependent metabolism, fatty acid metabolism, lipid metabolism, KRAS signaling pathway, etc. [13]. The result shows that the metabolic phenotypes in PDAC vary from patients because of the combined action of cell-autonomous pathways mediated by oncogenes, interactions with non-cancer cells and tumor microenvironment [14]. Metabolism-related genes, including CLUT, HK, MCT4, KRAS, participated in a series of physiological cascade reactions in PDAC, and lead to the development of PDAC. Because of the difficulty in quantifying the metabolism of patients in clinical practice and the rapid development of high throughput sequencing technology in precision medicine, we would like to use the metabolism-related genes to establish a model for predicting the prognosis of PDAC via multivariate COX analysis. Public databases ICGC, TCGA and GEO were involved in our studies.

Immune system is closely related with cancer development [16]. The immune system can recognize and eliminate tumor cells in the tumor microenvironment. However, in order to survive and grow, tumor cells can adopt different strategies to suppress the human immune system, which makes it unable to kill tumor cells normally. In this case, the tumor cells can survive in various stages of the anti-tumor immune response [11, 17]. This phenomenon is called tumor immune escape. Early analyses suggest that immunophenotype would be more powerful in predicting the prognosis of tumors than traditional AJCC stages [18–20]. CD8+ T cells was reported to be associated with the outcome of breast cancer [21], Foxp3+ regulatory T cells (Treg cells) was significantly associated with poor survival of majority of solid tumors, including cervical, renal, melanomas, and breast cancers [22]. In addition, previous studies found that subsets of TILs, especially CD3+, CD8+ and FoxP3+ T cells were strongly associated with long-term oncological outcomes in patients with PDAC [23]. Due to advances in understanding the cancer and its relationship with the immune system, more and more researchers are considering activating host immune defense as an effective anti-tumor response [24]. It has recently been proposed that increased metabolism was associated with immune evasion by tumor cells and is regulated by the changing tumor microenvironment (TME) [25]. For example, Hypoxia enhanced the glycolytic ability of tumor cells and increased the lactic acid growth rate. Producing large amounts of lactic acid acidifies the tumor microenvironment and affects the recognition and response of tumor cells by the immune system. Nutrient deficiency in the tumor microenvironment makes various cells compete with each other to meet their own needs, while immune cells are less adaptable to nutrient deficiency, thus forming an anti-tumor mechanism [26]. Previous studies found that metabolic pathway played an important role in the development of PDAC, and had essential effect on immune response in carcinogenesis of PDAC [27–29]. However, the dysregulated interaction between the immune system and metabolic pathways in PDAC has not been thoroughly studied. So, specific mechanisms between immune system and tumor metabolism in pancreatic cancer were explored through a series of studies in our study.

## Materials and methods

### Patients and clinical outcome assessment

This study utilized data from the First Affiliated Hospital of Wenzhou Medical University. We performed a retrospective study of PDAC patients from the First Affiliated Hospital of Wenzhou Medical University from January 2010 to January 2016. The inclusion criteria were as follows: (1) histopathological diagnosis or clinical diagnosis of PDAC; (2) all of the data were collected when patients diagnosed with PDAC firstly without any therapy. (3) Complete pathology, laboratory, and follow-up data. Patients with unknown included variables were excluded. The following demographic, clinical, and pathology data were used: T stage, N stage, M stage, tumor history, laboratory test results [age, gender, body mass index (BMI), TG, HDL-C, LDL, CHOL]. Metabolic syndrome was internationally defined as included more than three criteria: (1) BMI was greater than 25.0 kg/m^2^; (2) diagnosed with diabetes; (3) diagnosed with hypertension SBP/DBP > 140/90 mmHg; (4) blood HDL-C < 0.9 mmol/L, (5) blood TG > 1.7 mmol/L. Totally, there were 95 eligible cases selected in our study. All of these patients were followed up, and recurrent and dead patients were recorded during the follow-up. The time was cut-off until March 2021. The study protocols were approved by the Wenzhou Medical university Ethics Committee.

### Data download and preprocess

Transcriptomic data of PDAC patients with full clinical information from TCGA [30], ICGC [31], GSE28735, and GSE62452 were collected and analyzed in this study. Level 3 data of pancreatic cancer samples from TCGA data portal were downloaded from the website of National Cancer Institute (https://cancergenome.nih.gov/). Corresponding clinical information about these patients was obtained from the Cbioportal database (www.cbioportal.org/), including age, gender, AJCC stage, Histologic Grade, T-stage, N-stage, and follow-up information of PDAC. 65 patients from International Cancer Genome Consortium Pancreatic Cancer Australian (ICGC, PACA-AU) with RNA-sequencing raw counts and clinical survival data were included in the study. Raw read counts were downloaded through the Cbioportal database (www.cbioportal.org/). Normalized data of GSE28735 and GSE62452 were downloaded from GEO (https://www.ncbi.nlm.nih.gov/geo/) [32]. 125 patients with survival data were included in the survival analysis. GSE28735 and GSE62452 were performed by the same research team with identical Platform: GPL6244 [HuGene-1_0-st] Affymetrix Human Gene 1.0 ST Array [transcript (gene) version]. Partek Genomic Suite was used to remove the batch effect between two sets of data. Our analysis was performed based on this combined dataset.

### Construction of nomogram models

An OS nomogram was constructed based on the prognostic factors derived from multivariate Cox regression analysis to predict 1-, 3- and 5-year survival. Each patient could sum up each variable score and finally establish predictive measures of OS. The nomogram was generated using ggplot packages of R software. The calibration curve for predicting 1-, 3- and 5-year OS indicated that the nomogram-predicted survival closely corresponded with actual survival outcomes. The survival analysis were conducted using rms, survivalROC, survcomp and survival package. Hazard ratios (HRs) and 95% confidence intervals (CIs) were recorded.

### Model establishment and survival analyses

Patients with pancreatic cancer from TCGA databases were applied to identify a clinically translatable gene signature. A total of 1466 metabolic genes obtained from 70 KEGG metabolic gene sets [33] were evaluated by univariate Cox regression analysis. The most relevant 20 genes in prognosis were selected for further study. Multivariate stepwise Cox regression analysis was then used to identify the predictive metabolic-score model. The riskscore was calculated as follows:

$$\begin{aligned} {\text{Riskscore}} & = 0.91203*{\text{expression}}\;{\text{level}}\;{\text{of}}\;{\text{CA}}12 + 0.9*{\text{expression}}\;{\text{level}}\;{\text{of}}\;{\text{CDA}} + \left( { - 3.38811} \right)*{\text{expression}}\;{\text{level}}\;{\text{of}}\;{\text{DGKZ}} \\ & \quad + 6.76897*{\text{expression}}\;{\text{level}}\;{\text{of}}\;{\text{GMPS}} + \left( { - 6.09824} \right)*{\text{expression}}\;{\text{level}}\;{\text{of}}\;{\text{PI}}4{\text{KB}}{.} \\ \end{aligned}$$

PDAC patients were divided into a high-risk group and a low-risk group based on their median risk score. Survival analysis was applied with the R [34] package “survminer”, two-sided log-rank tests were applied to determine survival differences between high-risk patient groups and low-risk patient groups. The receiver operating characteristic (ROC), which was calculated by the R package of “survival ROC”, is used to evaluate the predicting power of our model. Another 65 PDAC patients from ICGC databases and 125 patients from GEO databases were used as two independent validation cohorts.

### Construction of random trees

Cox regression analysis was used to compare the importance of the clinical information and risk score. The results were shown by forest plot via the R package “forestplot”.

### Module determination

The top 25% of most variant genes were selected to construct a co-expression network with Weighted gene co-expression network analysis (WGCNA) [35]. The correlation between modules and clinical features was evaluated by Pearson correlation coefficients.

### Function analysis and Hub gene identification

To further demonstrate the mechanism underlying the module genes with correlative clinical factors. Genes in the interest module were further analyzed by Gene Ontology (GO) and Kyoto Encyclopedia of Genes and Genomes (KEGG) through R packages “ggplots2” and “clusterProfiler”. In addition, all Genes in the interest module were uploaded to the STRING database (https://string-db.org/cgi/input.pl) to construct the protein–protein network (PPI). The Cytohubba-plugin-based Cytoscape [36] was applied to analyze the network [37]. The top 20 high-degree genes were identified.

### GSEA analysis

GSEA is a functional annotation tool applied to understand the biological meaning of specified genes [38]. In our study, PDAC patients were firstly separated into the high-risk group or low-risk group based on their median risk score. Immune-related pathways between high-risk group and low-risk group were identified through the java software GSEA (http://www.broadinstitute.org/gsea).

### Assessment of immune infiltration

CIBERSORT is a gene expression-based deconvolution algorithm that uses gene expression signatures to estimate the immune composition of a tumor biopsy [39]. In our study, PDAC patients were divided into different groups according to their risk score. The infiltration of immune cells of each PDAC patient was calculated through CIBERSORT.

### Gene expression analysis

Gene expression in normal and tumor tissues was analyzed through a web server: CEPIA2 (http://gepia2.cancer-pku.cn/) [40]. 179 tumor tissue samples of PDAC from cancer genome mapping (TCGA) and 167 normal pancreatic tissue genotype tissue expression (GTEx) were included in our analysis.

### Survival analysis

The predictive performance of the hub genes in PDAC patients was further analyzed through the Kaplan–Meier survival analysis. The overall survival curves were generated using the R package “survminer”. Progression-Free survival (PFS) is defined as the time between the start of randomization and the progression of tumorigenesis (in any respect) or death (from any cause). Disease-Free survival (DFS) is defined as the time between the beginning of randomization and the recurrence of disease or death (from any cause).

### Cell lines

Human PC cell lines PANC-1 and Mia paca-2 were obtained the Cell Bank of China Academy of Sciences (Shanghai, China) and were cultured in DMEM medium (Gibco company, Cat#: 11966025) with 10% fetal bovine serum (Gibco company, Cat#: 10099141), 100 U/mL Penicillin, and 100 μg/mL Streptomycin (Gibco company, Cat#: 10378016). Cells were cultured in an incubator with 5% CO_2_ at 37 °C.

### CRISPR/Cas 9-mediated deletion of GMPS

The clustered regularly interspaced short palindromic repeats (CRISPR)/Cas9 system was used to knockdown GMPS. The CRISPR gene editing has been described previously [41]. Briefly, the oligos were designed based on information available at http://crispr.mit.edu and cloned into the lentiCRISPR/Cas9 vector (Add gene, Cat#: 49535) by following the Zhang laboratory’s protocol: (1) digest 5 μg of the lentiviral CRISPR plasmid with 3 μL BsmBI (NEB, Cat#: R0739) for 30 min at 37 °C; (2) phosphorylate and anneal each pair of oligos with T4 PNK Buffer (NEB) and T4 PNK (NEB, Cat#: M0201S) using the following parameters: 37 °C 30 min 95 °C 5 min and then ramp down to 25 °C at 5 °C/min; (3) dilute annealed and phosphorylated oligos from Step 2 at a 1:200 dilution into sterile water; (4) 50 ng digested lentiviral CRISPR plasmid from step 1, 1 μL diluted oligo duplex from Step 3, 5 μL 2× Quick Ligase Buffer (NEB), 1 μL Quick Ligase (NEB, Cat#: M2200S) and ddH2O were put together to set up the ligation reaction and incubated at room temperature for 10 min; (5) the plasmids of sgRNA-GMPS were transformed into Escherichia coli DH5alpha and were send out for sequencing (Shanghai Sunny Biotechnology Co, No. SH5338). The sgRNAs for GMPS were as follows:

- SgRNA-1-F 5′-CACCGGCCCCGATGGCTCTGTGCAA -3′
- SgRNA-1-R 5′-AAACTTGCACAGAGCCATCGGGGCC-3′
- SgRNA-2-F 5′-CACCGCTTTGAACAGATGATGAATA-3′
- SgRNA-2-R 5′-AAACTATTCATCATCTGTTCAAAGC-3′

After 72 h of infection with the lentiCRISPRv2 plasmid, PANC-1, and CFPAC-1, cells were selected by 10 μg/mL puromycin (Sigma, Cat#: P8833).

### Lentivirus infection for the construction of GMPS-overexpression

For overexpression of GMPS, pcDNA 3.1-GMPS (+) was constructed and used. Lentivirus was produced in 293T cells using the transfection reagent (QIAGEN, Cat#: 301425) as the manufacturer’s instructions. Supernatant containing virus was collected after 48 h transfection.

PANC-1 and CFPAC-1 cells were transfected at the confluence between 30 and 50% with Lipofectamine 3000 (Invitrogen Cat#: L3000008), and selected with 10 μg/mL Neomycin (Sigma, Cat#: 1405-10-3) after 48-h-transfection.

### Cell proliferation analyses and colony formation assay

According to the manufacturer’s instructions, cell growth was evaluated by the Cell Counting Kit-8 assay (Dojindo, Japan, Cat#: CK04). PANC-1 and CFPAC-1 were seeded in 96-well plates (2000 cells/well, respectively) in a final volume of 100 μL. After 0, 24, 48, and 72 h of their attachment, 10 μL of CCK-8 reagent was added to each well and incubated for 2 h at 37 °C in the cell incubator. The absorbance was measured at 450 nm. The assays were performed in triplicate. For colony formation assay, 5000 cells were seeded in 60 mm dishes for 14 days and were fixed with 4% paraformaldehyde for 15 min. Then, 0.1% crystal violet was added, and let the solution stand for another 15 min. Clone numbers were measured by microscopy, and pictures were obtained.

### Cell migration and invasion assays

According to the manufacturer’s instruction, the cell migration and invasion assays were performed by Transwell® chambers (8 μm pore size; Millipore, Cat#: PIEP12R48). For cell migration assay, 5 × 10^4^ cells were suspended in a serum-free medium and were placed in the top chambers. Then, a complete medium with 10% FBS was added into bottom chambers. The chambers were then cultured for 24 h at 37 °C in 5% CO_2_. Then, cells migrated through the Matrigel (Corning, Cat#: 356234) were fixed with 4% paraformaldehyde for 15 min followed by the addition of 0.1% crystal violet for another 15 min and stained with 0.1% crystal violet. Five fields of digital images were taken randomly, and the cells in each field were counted. For cell invasion assay, 5 × 10^4^ cells were plated in 200 μL of serum-free medium and were seeded in top chambers with Matrigel. The culture media with 10% FBS was added into lower chambers. After 24 h, cells invading the matrix were fixed, stained with 0.1% crystal violet, and counted under a microscope.

### Western blot assay and immunoprecipitation

The cells were collected and lysed on the ice with 1% SDS. Samples were separated by SDS-polyacrylamide gel electrophoresis and transferred to PVDF membrane (Millipore, Billerica, Cat#: ISEQ00010). Then, blots were blocked in 5% milk (5% low-fat milk powder in TBST), and then incubated with the appropriate primary antibody at 4 °C overnight. GAPDH was used as a loading control. The next day, blots were incubated with the secondary antibodies (1:5000) and labeled with horseradish peroxidase (HRP) for 1 h at room temperature. The Fusion FX7 ECL western blot system (Vilber Lourmat, France) was used to visualize the protein expression.

Rabbit GMPS (Cat#: 14602S), anti-rabbit antibodies (Cat#: 7074s) and anti-mouse (Cat#: 7076s) were purchased from Cell Signaling Technology. GAPDH (Cat#: sc-47724) was purchased from Santa-Cruz Biotechnology.

All primary antibodies were confirmed to be reactive only to manufacturer’s targets and used at 1:1000. Secondary antibodies were used at 1:5000.

### Statistical analysis

Metabolism-related gene sets were extracted from the Molecular Signatures Database v5.1 (MSigDB) (http://software.broadinstitute.org/gsea/downloads.jsp#msi-gdb), which contained a total of 1466 genes. Univariate Cox regression analysis and multivariate Cox regression analysis were applied by R package “coxph”. Random forest survival analysis was applied by SPSS.21. and demonstrated by R package “forestplot”. Survival analysis was applied with the R package “survminer”. The receiver operating characteristic (ROC) was calculated by the R package “survival ROC”. Nomogram was calculated and visualized through R package “rms”. All the data were analyzed by SPSS 21.0 Statistical program GraphPad Prism 6 software. *P* < 0.05 was considered as significant.

## Results

### Impact of metabolic syndrome on overall survival (OS) in patients with PDAC

A total of 95 PDAC patients from the First Affiliated Hospital of Wenzhou Medical University were involved in our study. As of March of 2021, 57 patients died during follow-up, none lost follow-up. The details of clinical information were shown in Additional file 1.

**Fig. 1:**
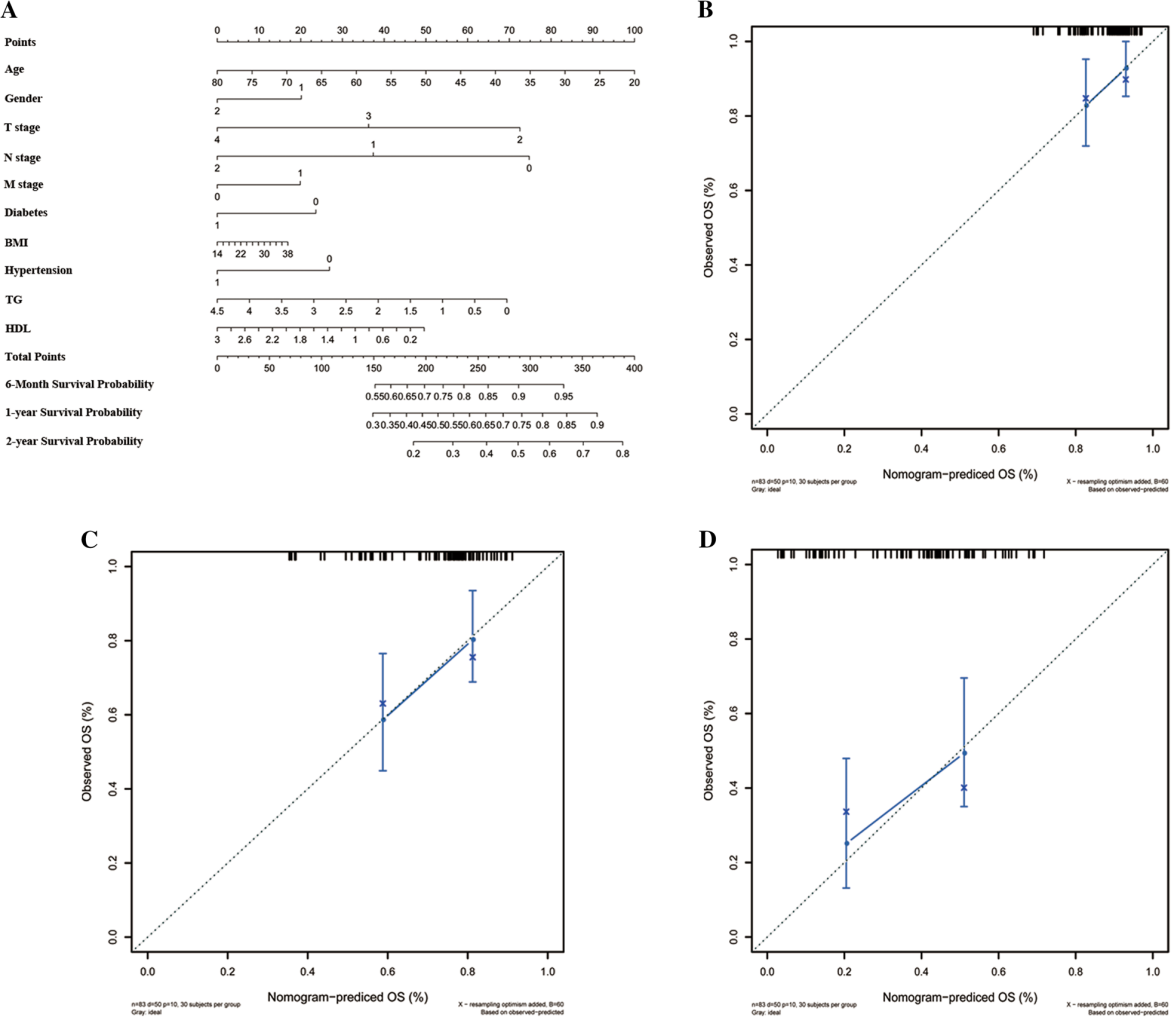
Nomogram developed to predict the overall survival of PDAC patients. **A** Nomogram developed by integrating metabolic syndrome and clinical pathological parameters for predicting 6-month and 1-, 2-year survival of PDAC patients; (**B**–**D**). Calibration curve for risk of 6-month and 1-, 2-year survival of metabolic syndrome

Metabolic syndrome is conferred as central obesity, dyslipidemia, hyperglycemia, insulin resistance and hypertension. Metabolic disorders were proven to be associated with the increased tumor risk. A nomogram was constructed to predict 6-month, 1- and 2-year overall survival of the PDAC. Total scores were summations of each variable based on the intersection of the vertical line. By using this nomogram, we could convert each clinical index to the corresponding point, and then calculate the total point, which was used to evaluate the 6-month, 1- and 2-year survival rate. We found that metabolic syndromes and together with other clinical factors played an important role in the prognosis of PDAC patients (Fig. 1A). Moreover, decision curve analysis showed the high accuracy of the predictive prognostic of MetS score for 6-month, 1- and 2-year OS possibility (Fig. 1B–D).

### Identification of metabolism-related signature in patients with PDAC

In order to systematically characterized the distinct molecular mechanism of MetS genes in PDAC patients, patients from the TCGA database were set as a training dataset and applied to establish the Metabolism-related Signature. A total of 1466 metabolism-related genes were applied to univariate Cox regression analysis. A list of 20 genes associated most with the prognosis was selected for further study (*P* < 0.0001). Through the Multivariate stepwise Cox regression analysis, a 5-gene prognosis model was successfully constructed. The riskscore of each patient was calculated as follows:

$$\begin{aligned} {\text{RiskScore}} & = 0.91203*{\text{expression}}\;{\text{level}}\;{\text{of}}\;{\text{CA}}12 + 0.9*{\text{expression}}\;{\text{level}}\;{\text{of}}\;{\text{CDA}} + \left( { - 3.38811} \right)*{\text{expression}}\;{\text{level}}\;{\text{of}}\;{\text{DGKZ}} \\ & \quad + 6.76897*{\text{expression}}\;{\text{level}}\;{\text{of}}\;{\text{GMPS}} + \left( { - 6.09824} \right)*{\text{expression}}\;{\text{level}}\;{\text{of}}\;{\text{PI}}4{\text{KB}}{.} \\ \end{aligned}$$

### Performance of the risk score in training dataset and validation dataset

**Fig. 2:**
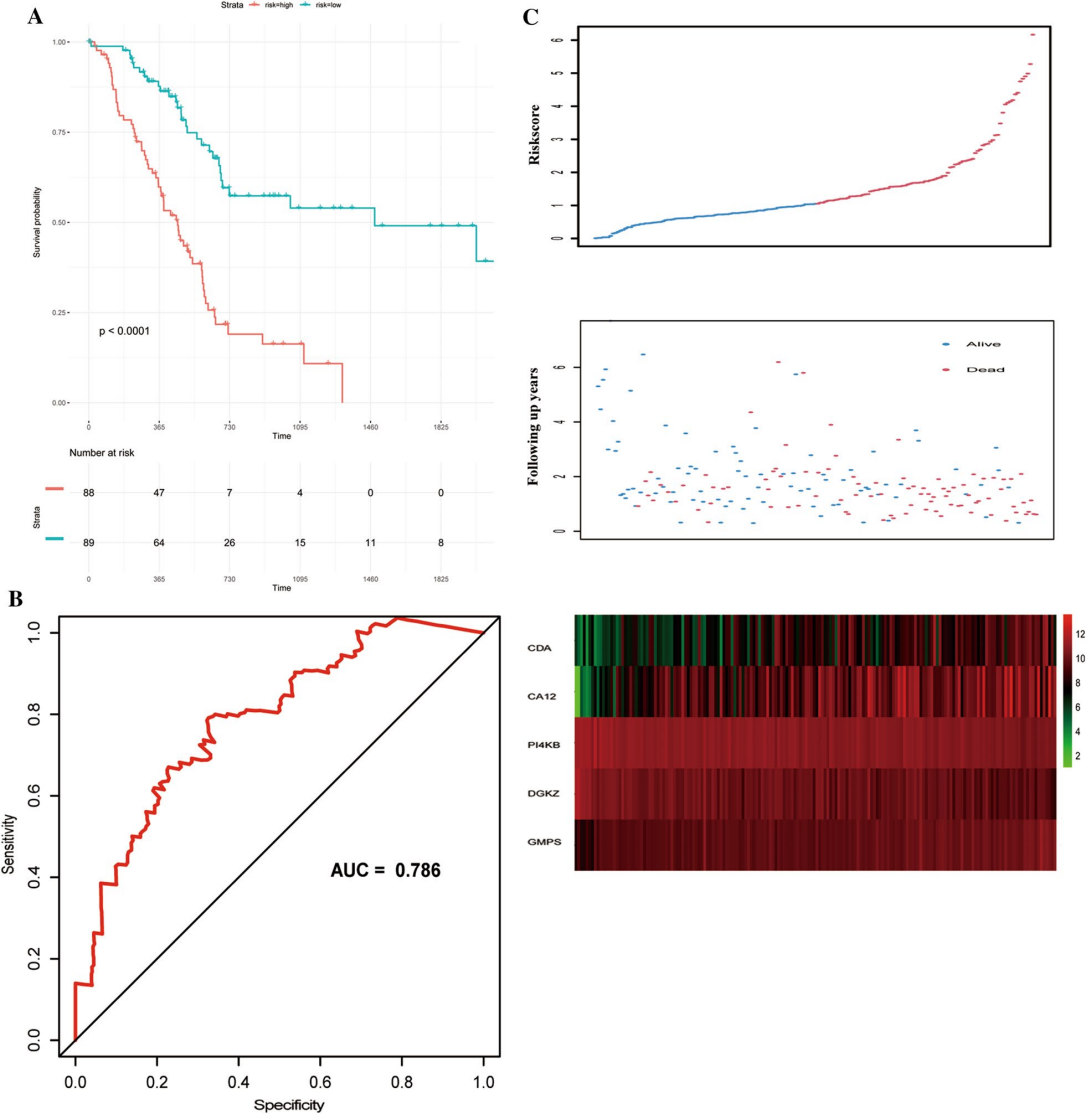
Performance of the prognostic model in the TCGA dataset. **A** Survival curve of overall survival between high-risk group and low-risk group in TCGA dataset. **B** Receiver operating characteristic (ROC) curves for 5-year survival in TCGA dataset. **C** Risk score distributions, survival status and expression profiles of the 5 genes of patients in TCGA dataset

Patients with PDAC were divided into a high-risk group (N = 88) and a low-risk group (N = 88) according to their median risk score. The performance of the risk score in predicting the prognosis of the PDAC patient was firstly verified in the training dataset. The survival analysis revealed that the PDAC patients with high-riskscore had a significantly shorter overall survival time than patients in low-riskscore group (*P* < 0.001, Fig. 2A). Receiver operating characteristic (ROC) analysis was used to describe the discrimination accuracy of our model. The area under the ROC curve (AUC) of the model was 0.786 in the TCGA data set, which indicates that the reliability of our PDAC’s prognosis model. (Fig. 2B). As Fig. 2C showed, as the riskscore increased, death toll increased and the follow-up time decreased. In addition, we found that the overall survival (OS), disease-free survival (DFS) and progression-free survival (DFS) were shortened in the PDAC patients in TCGA database with an increasing risk score (*P* < 0.00001, respectively) (Additional file 2: Fig. S1).

**Fig. 3:**
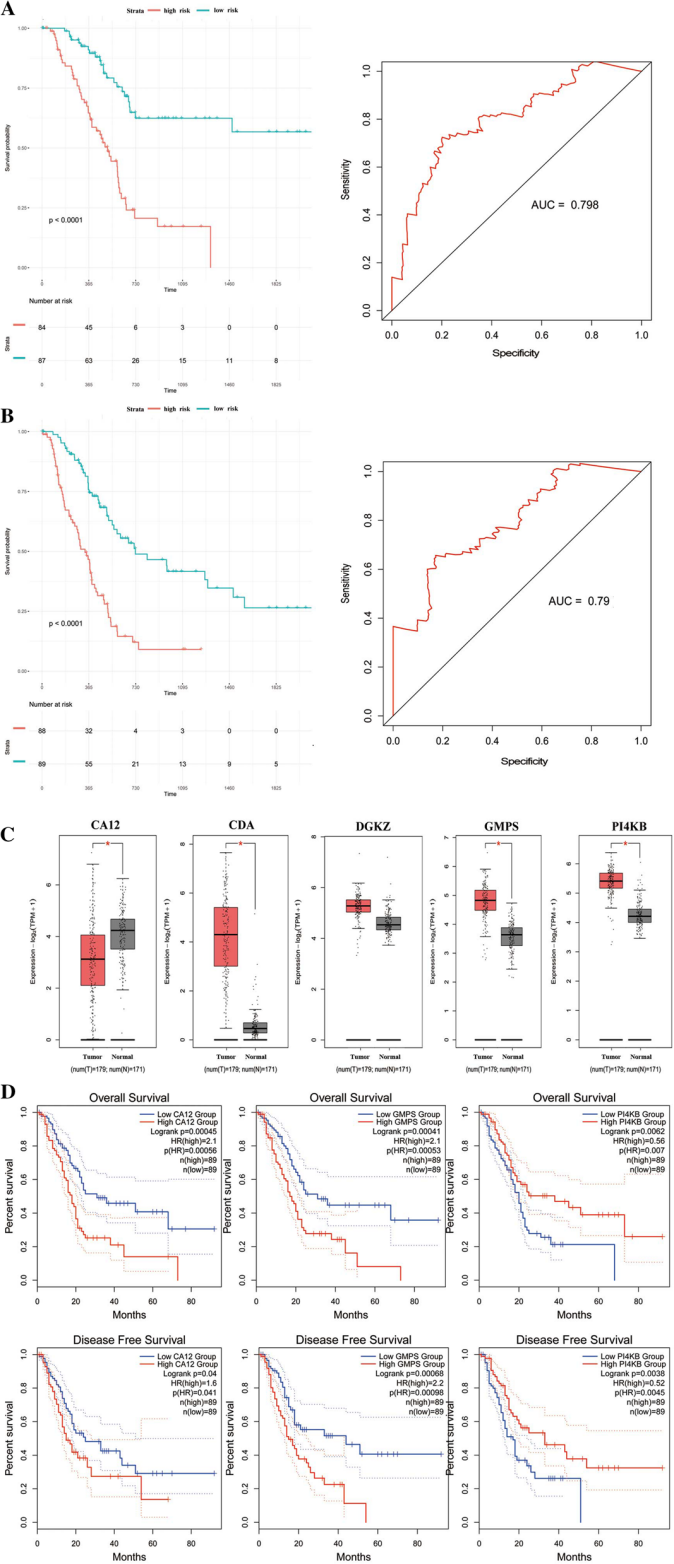
The performance of the prognostic model in prediction the PFS and DFS in TCGA dataset. **A**, **B** Survival curve of PFS and DFS between high-risk group and low-risk group in TCGA dataset and relative ROC curve. **C** Expression of CA12, CDA, DGKZ, GMPS and PI4KB in pancreatic cancer and normal pancreatic tissues. The red box represents tumor and grey box represents normal. **D** Survival analysis of CA12, CDA, DGKZ, GMPS and PI4KB in pancreatic cancer and normal pancreatic tissues

Besides, progression-free survival (PFS) and disease-free survival (DFS) of two groups were compared with each other respectively. The results showed that patients with high riskscore had a significantly shorter PFS, so as DFS, than the patients with low riskscore (*P* < 0.0001, respectively). The area under the ROC curve (AUC) for the model was 0.79 and 0.798, respectively (Fig. 3A, B). These results suggest that the risk score act as a strong prognostic indicator for PDAC patients. Risk score distributions, survival status and expression profiles of the 5 genes of patients in ICGC dataset and GEO dataset was shown in Additional file 3: Fig. S2.

Furthermore, the expression of the model's constituent genes between PDAC and normal tissue was investigated via CEPIA2, a web server. The results showed that the expressions of the CDA, GMPS, and PI4KB were significantly elevated in PDAC from the TCGA dataset compared with normal tissues from the GTEx dataset, while CA12 was downregulated in PDAC (Fig. 3C). Survival analysis showed that CA12, GMPS, and PI4KB were associated with the prognosis of PDAC (Fig. 3D).

Moreover, two independent validation cohorts were applied to evaluate the robustness of our model in PDAC patients. In the ICGC databases, based on the formula, PDAC patients were subdivided into high-risk and low-risk groups according to their median risk score. Consistent with training cohort results, we found that patients in the high-risk group had a significantly shorter OS than patients in the low-risk group (*P* = 0.034, Additional file 4: Fig. S3A). Then, this 5-gene signature was further applied to the GEO dataset. In agreement with the TCGA dataset and ICGC dataset, the survival analysis showed that patients with high riskscore had a worse prognosis than those with low riskscore (*P* = 0.005, Additional file 4: Fig. S3B).

### The nomogram prediction model

**Fig. 4:**
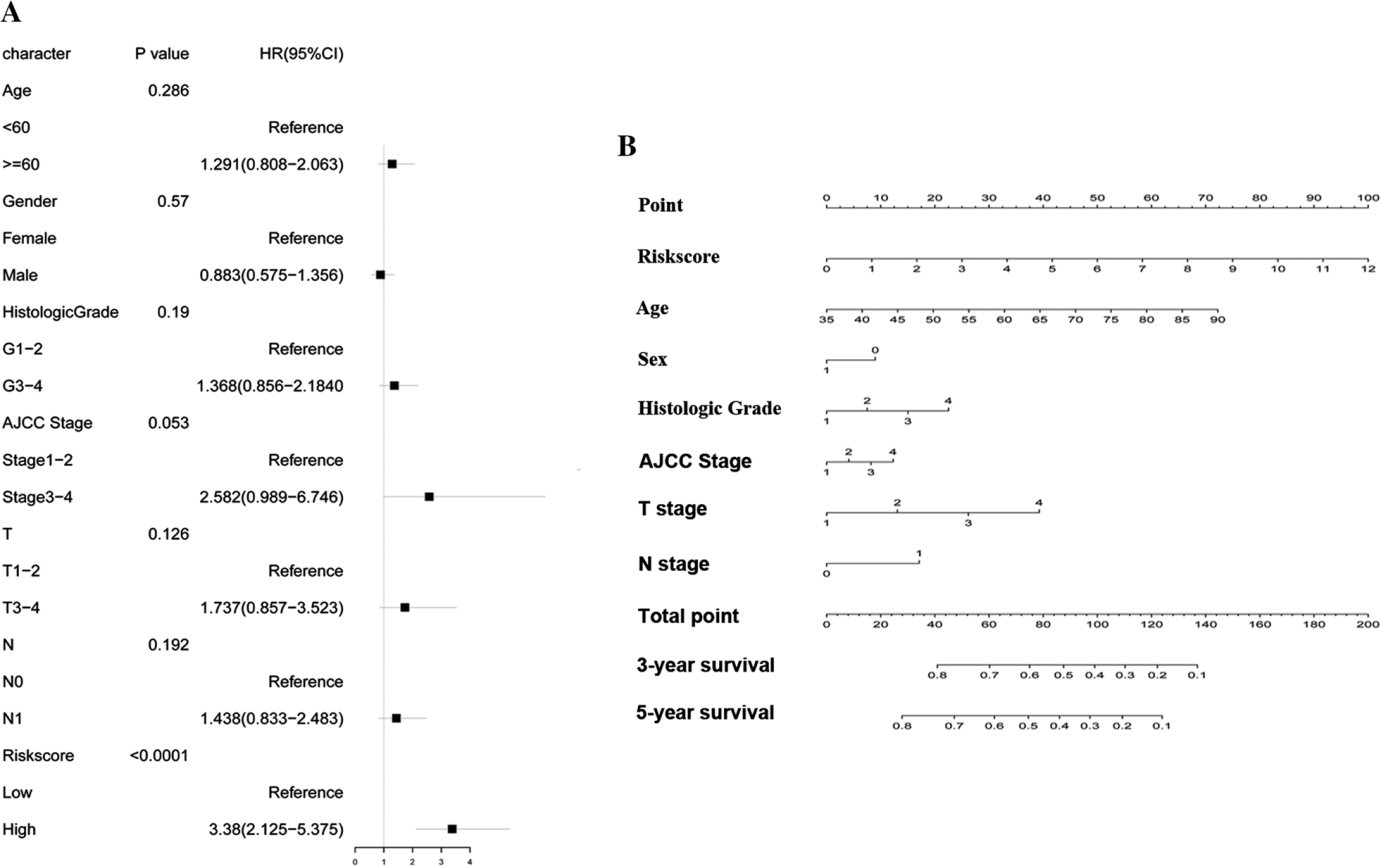
Confirmation of prognostic value of the prognostic model in TCGA dataset. **A** The clinical significance of risk score in TCGA dataset. *CI* confidence interval; HR. **B** Nomogram containing risk score with other clinical information was constructed

Multivariate Cox hazard analysis was performed to compare the robustness of risk score with other clinical information in predicting the prognosis of PDAC patients. our analysis contains clinicopathologic indicators including Riskscore, Age, Gender, Histologic Grade, AJCC stage, T stage, and N stage. As shown in Fig. 4A, Riskscore maintained independence from other clinical indicators in predicting the OS of PDAC patients and was the only factor that was remarkably correlated with the prognosis of PDAC patients in the TCGA dataset [*P* < 0.0001, HR (3.38 (2.125–5.375))]. According to the Cox regression, a nomogram was built to predict the prognosis of PDAC patients in clinical practice (Fig. 4B).

### Construction of a weighted correlation network and the identification of target module

**Fig. 5:**
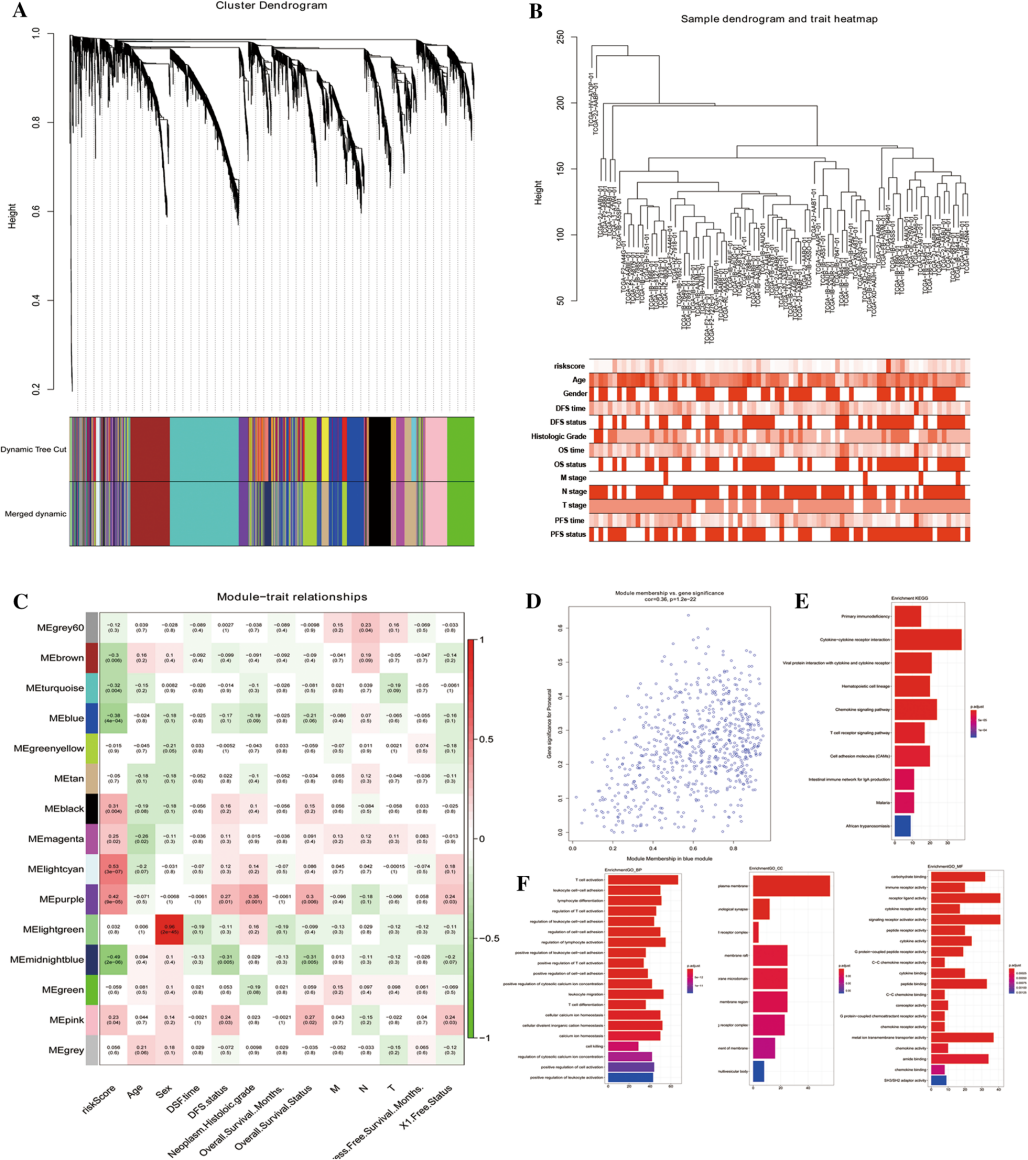
Weighted Gene Co-Expression Network Analysis Identifies risk score related module in patients with PDAC. **A** Dendrogram of genes clustered based on a dissimilarity measure (1-TOM). **B** The clustering was based on the expression data of expressed genes in PDAC patients. The color intensity was proportional to older age, male, longer survival time, higher histologic grade and T, N, M stage. **C** Heatmap of the correlation between module eigengenes and different clinical information of PDAC patients. **D** The scatter plot of the genes in blue modules and GO enrichment and KEGG pathway analysis of the blue module

The top 25% of most variant genes were selected to construct a co-expression network with Weighted gene co-expression network analysis (WGCNA) with WGCNA R package (Fig. 5A). Clinical data including age, gender, DFS time, DFS status, Histologic grade, OS time, OS status, T stage, N stage, M stage, and risk score were included in our research (Fig. 5B). The power of 8 (scale-free R2 = 0.88) was selected as the soft-thresholding. A total of 15 modules were identified among the patients with PDAC and assigned different colors. The association between each module and the clinical character was then analyzed. The results showed that the blue module (Correlation coefficient = − 0.38, *P* = 4e−04), lightcyan module (Correlation coefficient = 0.53, *P* = 3e−07), midnight blue module (Correlation coefficient = − 0.49, *P* = 2e−06) and purple module (Correlation coefficient = 0.42, *P* = 9e−05) were strongly correlated with the risk score of PDAC patients (Fig. 5C). Figure 5D showed a scatter plot of genes in blue modules.

### Function enrichment analysis

The target module genes were further analyzed by GO and KEGG to obtain insights into the function of genes in the hub module. GO analysis showed that the genes in the blue module were significantly enriched in T cell activation, leukocyte cell–cell adhesion in biological process (BP); external side of plasma membrane, immunological synapse, alpha–beta T cell receptor complex in Cellular Component (CC); carbohydrate-binding, immune receptor activity, receptor-ligand activity in Molecular Function (MF) (Fig. 5E). Moreover, hub genes were also highly represented in these top 5 KEGG pathways, including cytokine-cytokine receptor interaction, viral protein interaction with cytokine and cytokine receptor, hematopoietic cell lineage, chemokine signaling pathway, and T cell receptor signaling pathway (Fig. 5F).

### CIBERSORT analysis of tumor-infiltrating immune cells (TIICs) in pancreatic cancer

**Fig. 6:**
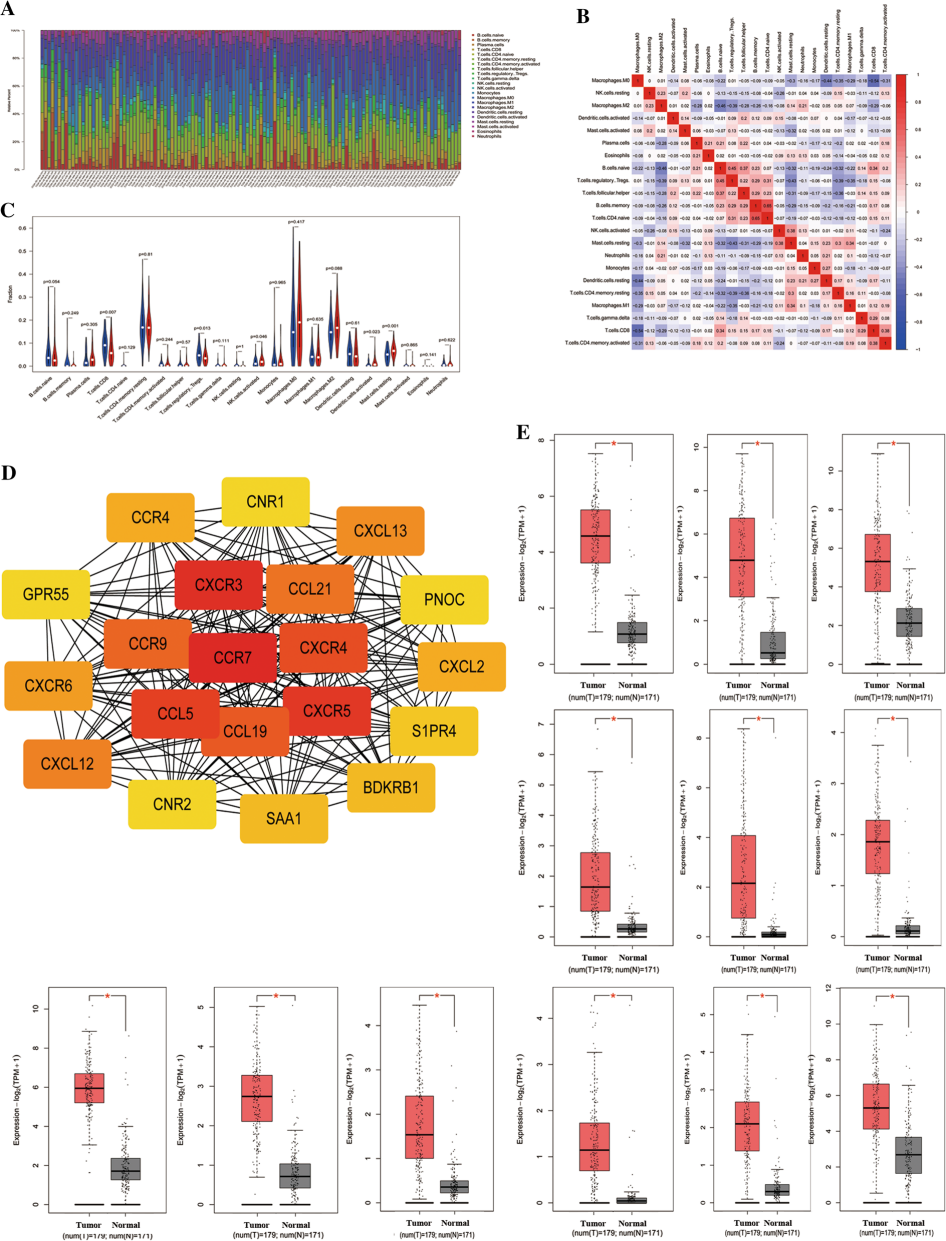
CIBERSORT analysis of tumor-infiltrating immune cells (TIICs) in pancreatic cancer. **A** The proportions of the 22 types TIICs in each PDAC patients. **B** Correlation matrix of all 22 immune cell proportions in TCGA dataset. **C** The proportions of the 22 types TIICs between the high- and low-risk groups. **D** The genes with a top 30 connectivity degree in the PPI network. The color intensity was proportional to higher connectivity degree. **E** Kaplan–Meier survival analysis of top 20 genes from blue module in PDAC patients

The function that chemokines and chemokine receptors can regulate the immune cells’ infiltration in tumors caught most of our attention. Furthermore, GO and KEGG analysis showed that genes in blue module enriched in the immune process, especially in T cells’ activities. Thus, we would like to explore whether the infiltration of immune cells was different between PDAC patients from high-risk and low-risk groups. CIBERSORT was applied to analyze the profile of tumor-infiltrating immune cells in PDAC. We divided the PDAC patients from TCGA into high-risk and low-risk group according to the median riskscore in our previous study. The differences in immune microenvironment between the high- and low-risk groups were investigated further. The TIICs composition of PDAC patients from two groups was analyzed by CIBERSORT. As shown in Fig. 6A, C, the intergroup proportions of the 22 types of TIICs were similar while the intragroup proportions were varied. A visualization of the relative proportions of 22 TIICs between the high-risk and low-risk groups was shown by violin diagram (Fig. 6C). The results showed that 2 TIICs (T cells CD8, T cells Regulatory) were in higher proportions in the low-risk group than those in the high-risk group, whereas 3 TIICs (Tregs NK cells Activated, Dendritic cells Activated, Mast cells Resting) were in higher proportions in the high-risk group (*P* < 0.05, respectively). Moreover, the two most common TIICs in PDAC tissues were B and T lymphocytes, accounting for approximately 50% of all TIICs. Figure 6B showed the correlation between each TIIC.

### Hub gene identification

Our study identified 693 genes in the blue module that correlated highly with the risk score of PDAC patients. Furthermore, we uploaded all genes in the blue module to the STRING database to construct a network of protein–protein interactions (PPI). In the PPI network, genes with a top 20 connectivity degrees were also defined as hub genes (Fig. 6D). The expressions of these 20 genes between pancreatic tumor and normal tissue was investigated via CEPIA2, a web server. The results showed that expressions of CCR7, CXCR3, CXCR5, CCL5, CXCR4, CCL19, CCL21, CXCL13, CXCR6, SAA1, S1PR4, PONC were significantly elevated in pancreatic tumor tissue from the TCGA dataset compared with normal tissues from the GTEx dataset. (Fig. 6E). In addition, the hub genes which expressed differently between pancreatic tumor tissue.

### Gene set enrichment analysis of the immune status between high-risk and low‑risk group

**Fig. 7:**
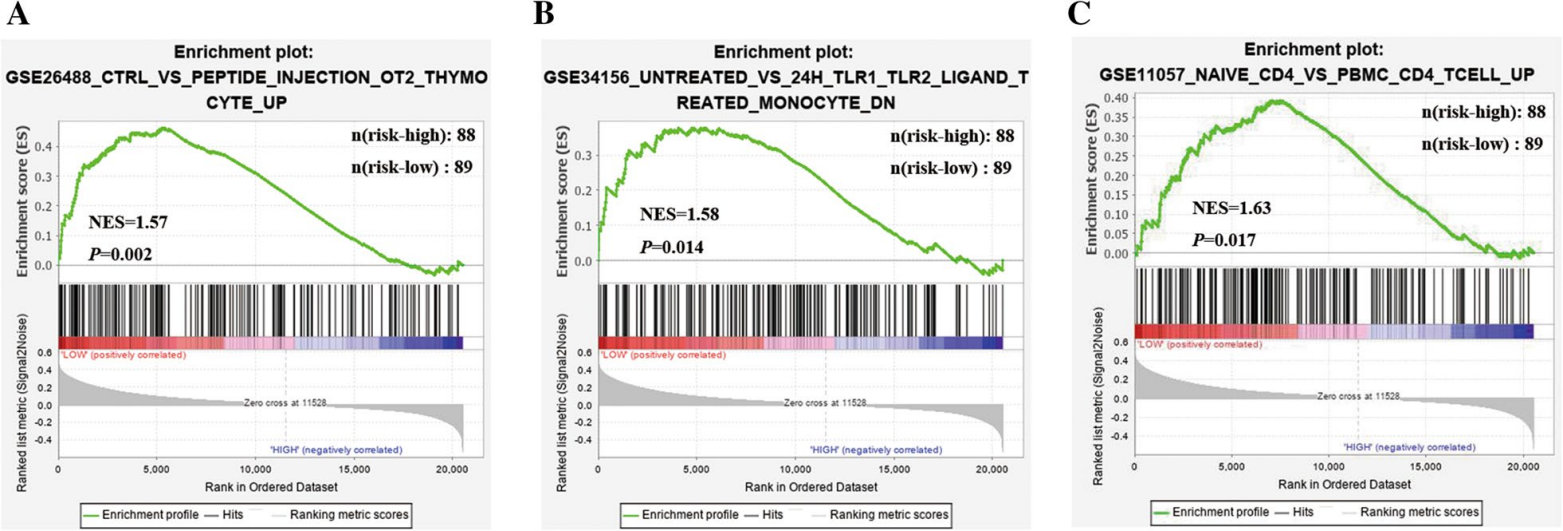
GSEA analysis of the immune status between high-risk and low‑risk group

GSEA analysis was applied to explore the potential Immune-related signaling pathways between high-risk and low-risk groups in TCGA dataset (Fig. 7A–C). Immunologic signature gene sets in Molecular Signatures Database (MSigDB) which contained 5219 gene sets were applied in our study. GSEA revealed that low riskscore was significantly associated with peptide injection OT2 thymocyte up (NES = 1.57, *P* = 0.002), 24H TLR1 TLR2 Ligand treated monocyte (NES = 1.58, *P* = 0.014) and PBMC CD4 T cell up (NES = 1.63, *P* = 0.017). The results elucidated that immune-related responses and processes played an essential role in the metabolism of PDAC patients.

### Knockdown of GMPS can significantly represses the proliferation and migration ability of pancreatic cancer cells

**Fig. 8:**
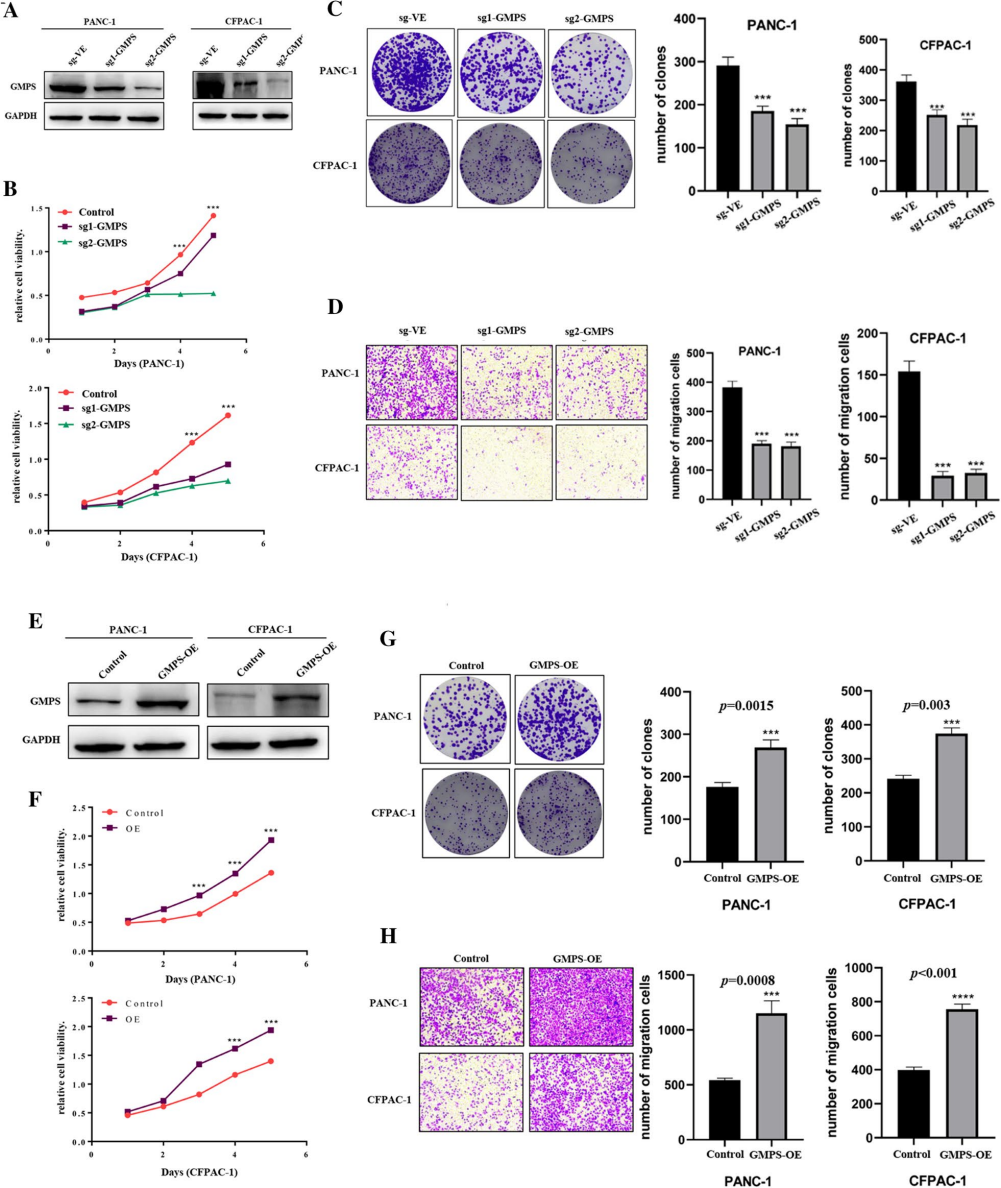
the performance of GMPS in PDAC cells in vitro. **A** GMPS knockdown was confirmed using western blot. **B**–**D** Knockdown of GMPS in PANC-1 and CFPAC-1 inhibited the proliferation and migration of tumor cells. **E** The expression of GMPS was confirmed using western blot. **F**–**H** Overexpression of GMPS in PANC-1 and CFPAC-1 enhanced the proliferation and migration of tumor cells

To evaluate the potential function of Guanosine Monophosphate Synthetase (GMPS) in PDAC, we knocked down GMPS in PANC-1 and CFPAC-1 cells through the CRISPR-Cas9 system. The efficiency of GMPS knockdown was verified by western blot (Fig. 8A). Moreover, the viability of cells was measured by Cell Counting Kit-8 (CCK-8) assay, and the result showed that GMPS knockdown significantly compromised the growth rate of PDAC cells (Fig. 8B). Similarly, the colony formation assay revealed that the GMPS knockdown markedly reduced the number of clones after 14 days of cultivation, compared with the control (*P* < 0.001) (Fig. 8C). Together, these results suggest that GMPS plays a positive role in the proliferation of PDAC cells in vitro.

Furthermore, Transwell assays were used to assess the migration and invasion of PC cells. The results showed that the motilities of PANC-1 and CFPAC-1 were significantly affected by the expression level of GMPS. The migration rate of cells with GMPS depletion was much lower than the control cells (Fig. 8D). These results demonstrated that GMPS depletion inhibited the migration of PDAC cells.

### Overexpression of GMPS significantly enhances the proliferation and migration of pancreatic cancer cells

To further assess the oncogenic role of GMPS, GMPS was overexpressed in the pancreatic cancer cell lines PANC-1 and CFPAC-1. Western blot analysis confirmed a significant increase in GMPS expression in PANC-1-GMPS and CFPAC-1-GMPS cells relative to the expression of GMPS in control cells (Fig. 8E). CCK-8 assay showed that the overexpression of GMPS increased viability of PANC-1 and CFPAC-1 cells (Fig. 8F). Moreover, the colony-forming assay showed that PANC-1 and CFPAC-1 cells with GMPS overexpression demonstrated a considerable growth advantage relative to the respective control cells (Fig. 8G). Meanwhile, a distinctly higher migration rate was observed in GMPS overexpression cell lines than in the control cells through Transwell assay (Fig. 8H). Thus, GMPS overexpression significantly enhances the proliferation and migration ability of pancreatic cancer cells in vitro.

## Discussion

Pancreatic cancer is one of the deadliest cancers worldwide with a 5-year survival rate dismal at ~ 8% [42]. The tumor occurrence is a progressive process under the joint action of internal and external pathogenic factors, such as environmental factors, genetic factors, dietary habits, etc. PDAC is a notable characteristic of dense stroma, with appropriate being up to 90% of the tumor volume, which contributes to lack of vascularization and hypoxia [43]. The hypoxia in the tumor microenvironment causes cancer cells to undergo metabolic stress and nutrient deprivation [44]. As a result, PDAC tumor cells reform to the so-called “metabolic reprogramming”, an updated hallmark of cancer. In recent years, more and more scientists have focused their attention on the metabolic changes of tumors. Unlike normal cells, PDAC cells have high glycolysis levels, even in the presence of oxygen and reduced mitochondrial function, leading to power themselves through aerobic glycolysis, also called the “Warburg effect” [45]. On the other hand, cancer cells induce oxidative stress in the neighboring stromal cells by secreting ROS, triggering aerobic glycolysis, and production of high energy metabolites, especially lactate and pyruvate, which is also called the “reverse Warburg effect” [46]. In a word, tumor cells increase glycolysis and glucose transport, high glutamine consumption, lipid, and amino acid biosynthesis to maintain the homeostasis. The differences in metabolism in PDAC have received renewed interest since altered homeostasis has been identified as a contributing factor to PADC progression. However, its underlying mechanisms remain not completely understood.

Our study integrated the metabolism-transcriptomics approach revealed that PDAC tissues exhibit a reprogramming of metabolism in association with an altered expression of metabolism-associated genes. We constructed on metabolism-related gene signature to predict the prognosis of pancreatic cancer based on TCGA databases and was further validated in the ICGC database and the GEO database. The results showed that PDAC patients with high riskscore would have a worse prognosis than patients with low riskscore. Our model contained five metabolism-related genes: CA12, CDA, DGKZ, GMPS, PI4KB. Carbonic anhydrase 12 (CA12) encodes zinc metalloenzyme, which belongs to the family of Carbonic anhydrases (CAs) that catalyze the reversible hydration of carbon dioxide. They are involved in a variety of biological processes, including salivary and gastric acid formation, respiration, bone resorption, calcification, etc. Previous studies have reported that CA12 is highly expressed in many human tumors and is related to the prognosis of patients [47, 48]. Narasimha Rao Uda etc., found that blocking the enzymatic activity of Carbonic Anhydrase 12 would decrease tumor proliferation, and CA12 would be a novel therapy target for CA12-positive tumor [49]. CA12 is overexpressed in breast tumor tissues than normal breast tissues and is significantly associated with breast cancer prognosis [50]. However, the research about CA12 and the progression of PDAC have not been reported. Cytidine deaminase (CDA) is reported to participate in pyrimidine salvaging. Currently, most of the studies focus on the role of CA12 in blood tumors. The silence of CDA in leukemia would inhibit tumor growth and promote cell apoptosis in Chronic Myeloid Leukemia (CML) cells [51]. Further study demonstrated that the prognosis of acute myeloid leukemia (AML) with the treatment of Cytarabine was strongly related to the expression of CDA. Patients with lower CDA activity would receive a higher response rate and better prognosis [52]. Moreover, CDA is reported to be overexpressed in pancreatic cancer and closely associated with the effect of Gemcitabine in PDAC [53, 54]. Based on genomic approaches, scientists found that CDA participated in the conversion of 5hmdC and 5fdC, leading to the accumulation of DNA damage, and resulted in the death of cells [55]. DGKZ is reported to act as an oncogene in osteosarcoma (OS) and is correlated with poor prognoses of OS patients, but the relationship between DGKZ and PDAC is lacking. The research focuses on PI4KB and tumor is lacking. The function of PI4KB in PDAC requires further studies. Similarly, previous studies have demonstrated that GMPS played an important role in the progression of ovarian cancer [56], hepatocellular carcinoma [57], myeloid [58] etc. The research focused on the role of GMPS in PDAC is rare. We found that the GMPS acted as an oncogene in PDAC. In the future, more time will be spent on the role and the specific mechanism of GMPS in PDAC. So far, we established a signature that could successfully predict the survival of PDAC. As is known to all, a single gene cannot accurately predict the outcome of PDAC patients, and we believe that the combination of these five genes based on the multivariate Cox analysis could enhance the sensitivity and specificity in predicting the outcome of PDAC patients and worth popularizing in clinic.

Moreover, the results of the forest plot showed that our signature is more powerful in predicting the prognosis of PDAC patients than other classic clinical characters. Our signature, which combined with clinical characters including, age, gender, AJCC stage, etc., made the model applicable in clinical practice. Our model provides new ideas for the diagnosis and treatment of pancreatic cancer in clinical practice.

Previous studies have demonstrated that the interaction between the immune system and tumor metabolism plays an important role in tumorigenicity and progression of pancreatic cancer [59]. Thus, we applied WGCNA to find potential immune-related genes associated with the riskscore of PDAC patients. The results showed that the blue module was significantly associated with riskscore of PDAC patients. The genes in this module were enriched in the immune-related signaling pathway. Then a total of 20 hub genes of the blue module were found through the construction of the PPI network. The result showed that CCR7, CXCR3, CXCR4, CXCR5, CXCR6, CXCL13, CCL5, CCL19, CCL21, SAA1, S1PR4, PONC were significantly elevated in pancreatic tumor tissue from TCGA datasets compared with normal tissues from GTEx datasets. We found that most of these genes belong to the chemokines and chemokine receptors family. In recent years, the role of chemokines and their receptor families in tumor development has attracted great attention [54–61]. Various evidence indicated that they were significantly associated with tumor progression. Tumor-related chemokines can not only promote the proliferation and inhibit the apoptosis of tumor cells, but also control the migration, angiogenesis of tumor cells, regulate the infiltration of immune cells in tumors, and participate in the selective metastasis of tumor cells. Through the presence of cytokines, monocytic cells were recruited to the tumor microenvironment and become tumor-associated macrophages (TAMs) [62]. Moreover, TAMs can mediate immunosuppression and angiogenesis and promote tumor progression by releasing cytokines, which triggers waterfall response for TAMs recruitment. Moreover, C-X-C motif chemokine receptors also contribute to Gemcitabine resistance, and combination with a CXCR4 antagonist (AMD3100) or hedgehog inhibitor (GDC-0449) with gemcitabine inhibit the growth of orthotopic pancreatic tumor-bearing mice [63].

The function that chemokines and chemokine receptors can regulate the infiltration of immune cells in tumors caught most of our attention. Furthermore, GO and KEGG analyses showed that genes in the blue module enriched in immune process, especially in T cells’ activities. Thus, we would like to explore whether the infiltration of immune cell was different between PDAC patients from high-risk group and low-risk group. CIBERSORT was applied to analyze the profile of tumor-infiltrating immune cells in PDAC. The results showed that that 2 TIICs (T cells CD8 and T cells regulatory) were in higher proportions in the low-risk group than those in the high-risk group, whereas 3 TIICs (Tregs NK cells activated, Dendritic cells activated and Mast cells resting) were in higher proportions in the high-risk group than those in the low-risk group. CD8^+^ T cells are the most virulent of T cells, and the number of CD8+ T cells around the tumor directly determines the damage to the tumor. The researchers found that renal carcinoma patients with less than 2.2% of CD8+ T cells had a four-fold higher risk of disease progression after surgery [64]. During the process of tumor immunity, besides T cells, NK cells, Dendritic cells, Mast cells also play critical roles. NK cells are regulatory cells which can shape the anti-tumor immune response by reciprocal interactions with dendritic cells, macrophages, T cells, and endothelial cells through a combination of cell surface receptors and secreted cytokines [65, 66]. PDAC has recently been found to impair NK cell tumor cell recognition and function by the regulation of several mediators, including transforming growth factor beta (TGF-β), interleukin (IL)-10, indoleamine 2,3-dioxygenase (IDO), and matrix metalloproteinases (MMPs) [67]. Although it shows an extremely low frequency of NK cell infiltration in the microenvironment, it undertakes the function in anti-tumor immune responses in PDAC. Dendritic cells played a critical role in the T-cell-mediated tumor immunity, which transports tumor antigens to T cell and finally activates cytotoxic T lymphocytes [68, 69]. Since the 1990s, growing research has set dendritic cells as a novel therapy target for cancer treatments and made gratifying progress [70]. As for Mast cells, previous studies have demonstrated that mast cells play a positive and negative role in tumor development [71] and act as a new therapeutic target in tumor treatment [72]. As mentioned above, targeting the metabolic aberrations to reprogram the metabolism in immune cells might lead to discovering novel therapeutic strategies.

However, firstly, this study was a monocenter prospective research, some selection, calculation bias and deviations were unavoidable. The outcomes should be validated by multicenter prospective information. Moreover, we have found that the expression of GMPS was higher in PDAC patients compared with normal patients and the expression of GMPS was associated with the outcome of PDAC patients. Further researches demonstrated that GMPS played as an oncogene in PDAC, it promoted proliferation and metastasis of PDAC cells. However, the relationship between GMPS and immunity system in PDAC cell is still unclear and more experiments are needed in the future. If possible, further experiments could be performance in vivo and in vitro to verify these results.

In conclusion, we established a metabolism-related signature to predict the prognosis of PDAC patients based on TCGA databases and was validated in ICGC databases and GEO databases. Future studies demonstrated that different of tumor infiltration of immune cells between high-risk and low-risk groups might cause the different prognoses of PDAC patients. However, experimental research on the mechanism between tumor-infiltration of immune cells and PDAC are still needed in the future.

## Supplementary Information

**Additional file 1:** The clinical information of 95 PDAC patients from the First Affiliated Hospital of Wenzhou Medical University.

**Additional file 2: Figure S1.** The overall survival (OS), disease-free survival (DFS) and progression-free survival (DFS) were shortened in the PDAC patients in TCGA database with an increasing risk score (P < 0.00001, respectively). (The patients were ranked based on the riskscore and then divided into four groups: Group 1: top 25% of the risk score; Group 2: top 26–50% of the risk score; Group 3: top 51–75% of the risk score; Group 4: last 76–100% of the risk score).

**Additional file 3: Figure S2.** Risk score distributions, survival status and expression profiles of the 5 genes of patients in ICGC dataset and GEO dataset, respectively.

**Additional file 4: Figure S3.** Performance of the prognostic model in ICGC dataset and GEO dataset. (A) Survival curve of overall survival between high-risk group and low-risk group in the ICGC dataset. (B) Survival curve of overall survival between high-risk group and low-risk group in the GEO dataset.

**Additional file 5: Figure S4.** GSEA found that the genes in low-subgroup were enriched in KEGG TPYE II DIABETES MELLITUS compared with those in high-subgroup (NES = 1.53, P = 0.028).

## Data Availability

The datasets used and/or analyzed during the current study are available from the TCGA, ICGC and GEO database and the First Affiliated Hospital of Wenzhou Medical University.
