# Supplementary figures and images for: Metabolic syndrome related gene signature predicts the prognosis of patients with pancreatic ductal carcinoma. A novel link between metabolic dysregulation and pancreatic ductal carcinoma
Source: Cancer Cell Int. 2021 Dec 20;21:698. doi: 10.1186/s12935-021-02378-w (PMC8690436; doi:10.1186/s12935-021-02378-w)

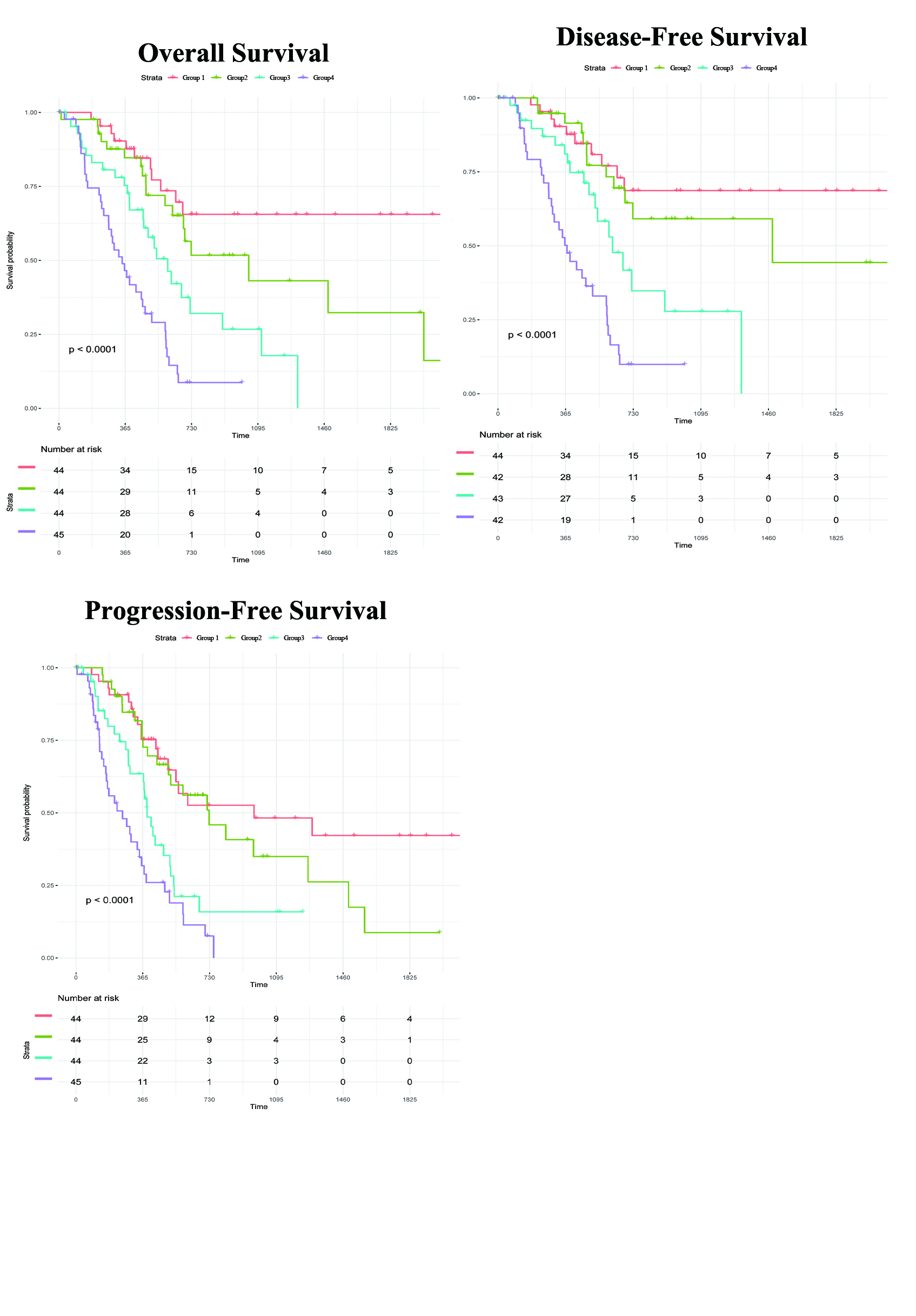

Supplement: Supplementary file 2 — Additional file 2: Figure S1. The overall survival (OS), disease-free survival (DFS) and progression-free survival (DFS) were shortened in the PDAC patients in TCGA database with an increasing risk score (P < 0.00001, respectively). (The patients were ranked based on the riskscore and then divided into four groups: Group 1: top 25% of the risk score; Group 2: top 26–50% of the risk score; Group 3: top 51–75% of the risk score; Group 4: last 76–100% of the risk score). [file 12935_2021_2378_MOESM2_ESM.tif]

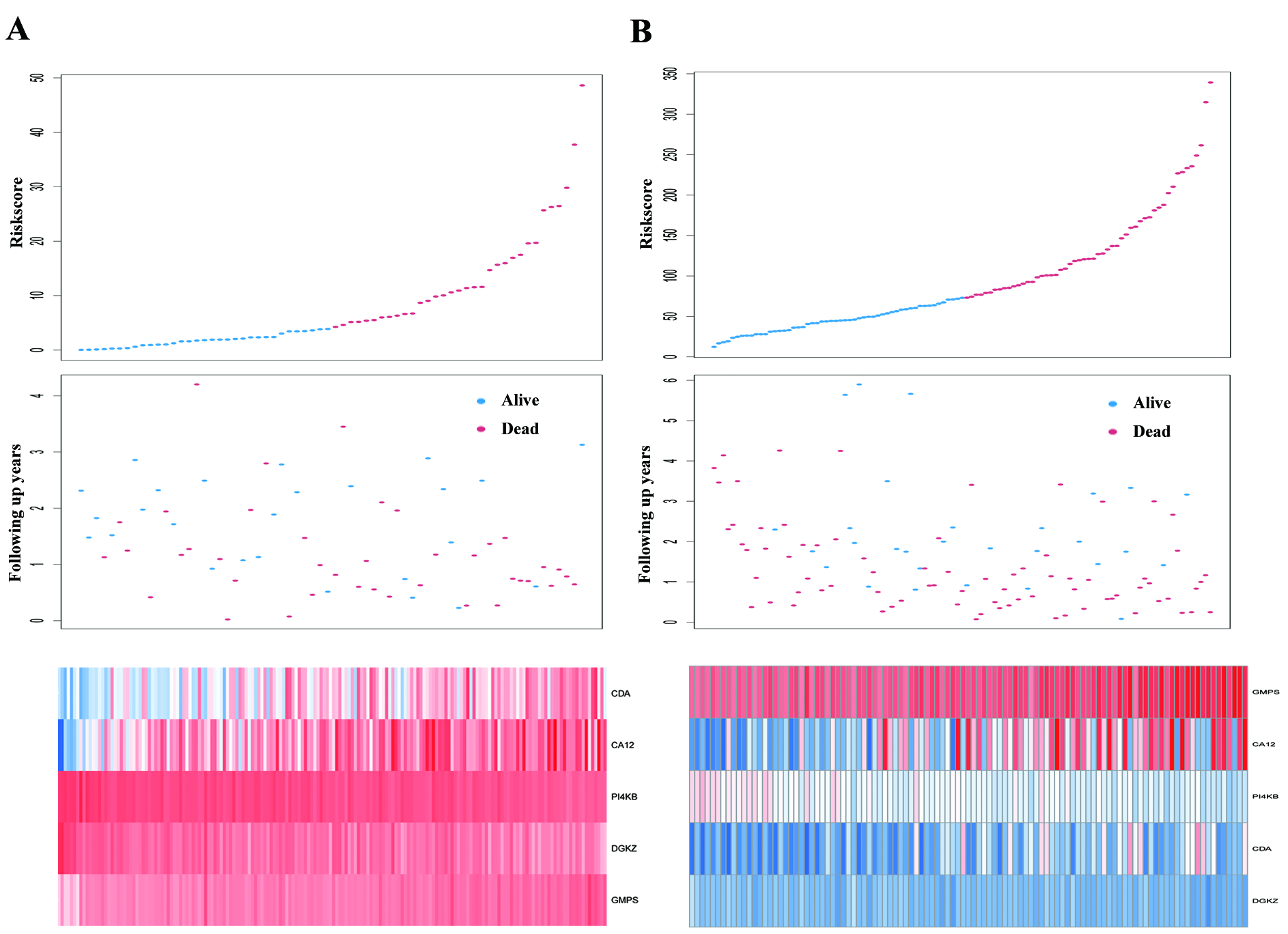

Supplement: Supplementary file 3 — Additional file 3: Figure S2. Risk score distributions, survival status and expression profiles of the 5 genes of patients in ICGC dataset and GEO dataset, respectively. [file 12935_2021_2378_MOESM3_ESM.tif]

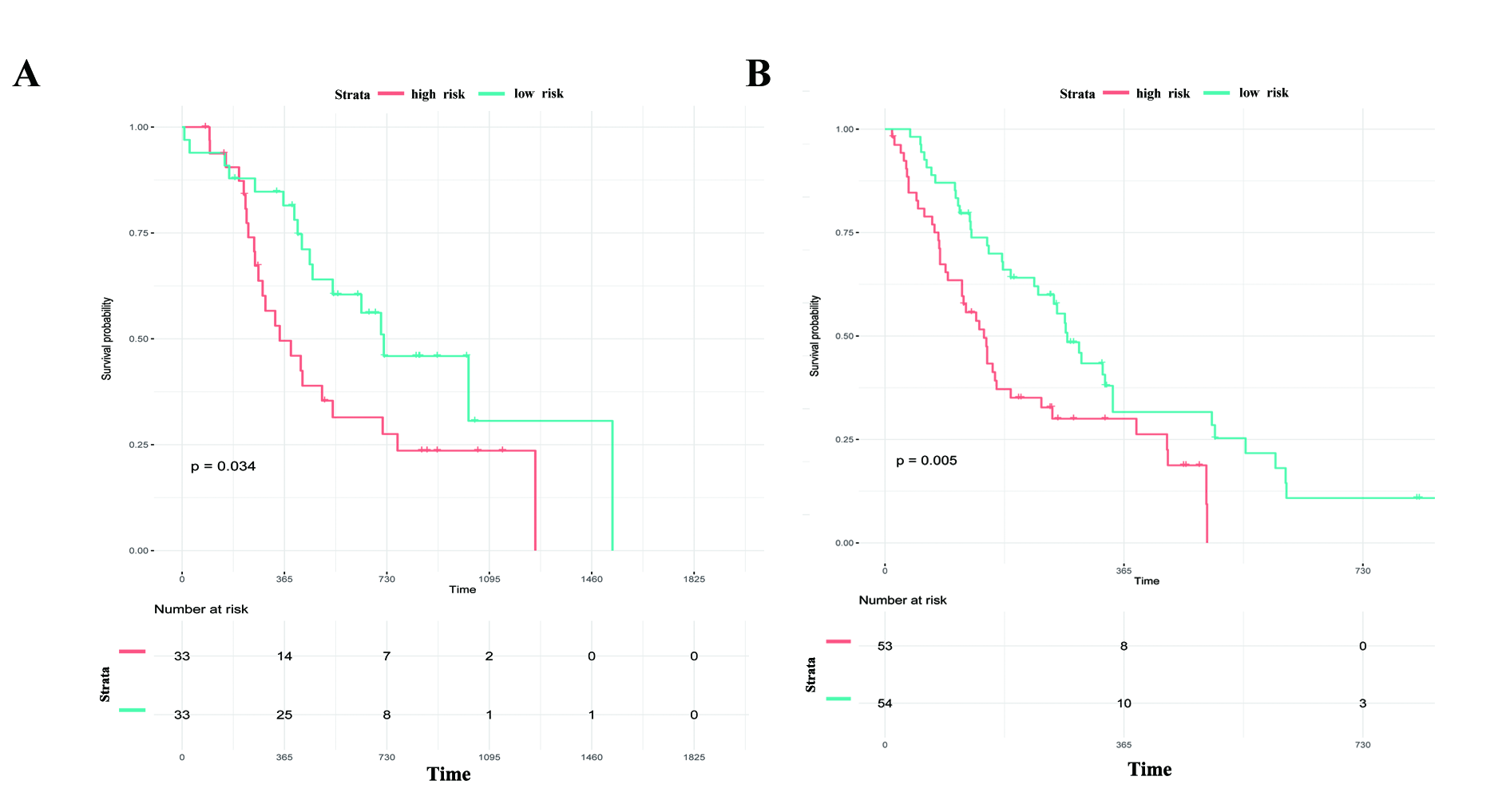

Supplement: Supplementary file 4 — Additional file 4: Figure S3. Performance of the prognostic model in ICGC dataset and GEO dataset. (A) Survival curve of overall survival between high-risk group and low-risk group in the ICGC dataset. (B) Survival curve of overall survival between high-risk group and low-risk group in the GEO dataset. [file 12935_2021_2378_MOESM4_ESM.tif]

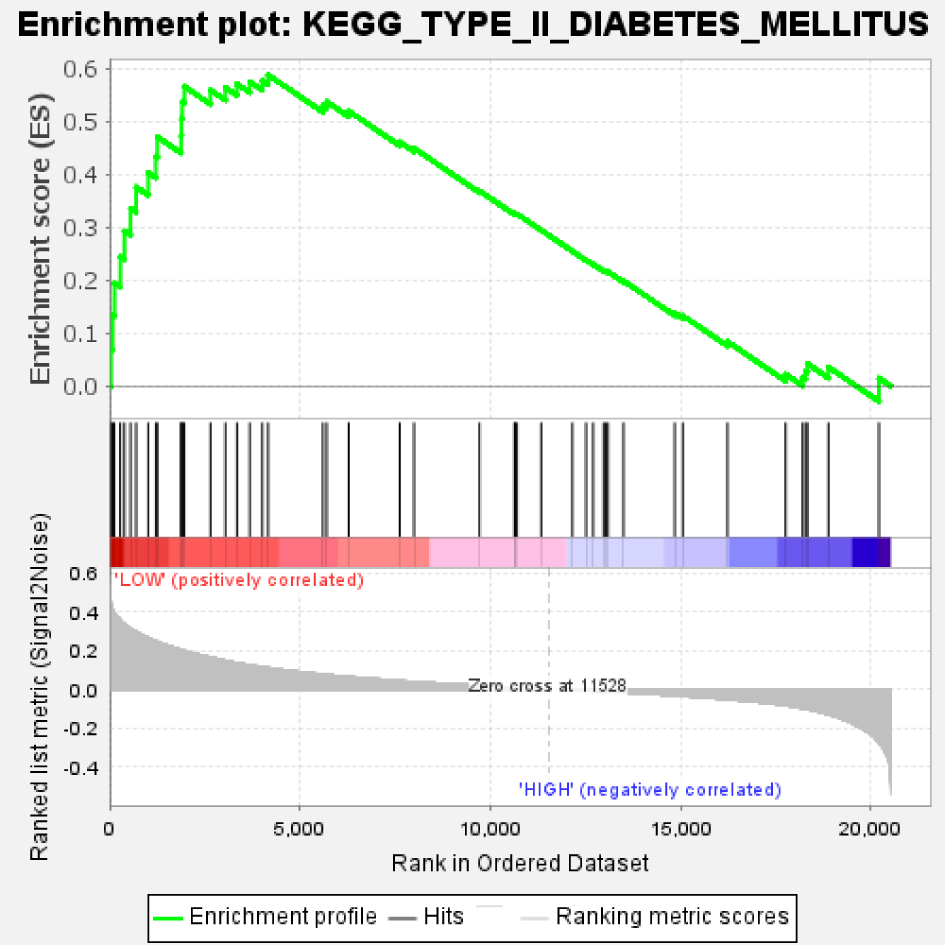

Supplement: Supplementary file 5 — Additional file 5: Figure S4. GSEA found that the genes in low-subgroup were enriched in KEGG TPYE II DIABETES MELLITUS compared with those in high-subgroup (NES = 1.53, P = 0.028). [file 12935_2021_2378_MOESM5_ESM.tif]
